# Evaluate Air Pollution by Promethee Ranking in Yangtze River Delta of China

**DOI:** 10.3390/ijerph17020587

**Published:** 2020-01-16

**Authors:** Xiaobing Yu, Chenliang Li, Hong Chen, Zhonghui Ji

**Affiliations:** 1 School of Management Science and Engineering, Nanjing University of Information Science & Technology, Nanjing 210044, China; 20191224003@nuist.edu.cn (C.L.); 20181224001@nuist.edu.cn (H.C.); zhonghuiji@mail.bnu.edu.cn (Z.J.); 2 Collaborative Innovation Center on Forecast and Evaluation of Meteorological Disasters, Nanjing University of Information Science & Technology, Nanjing 210044, China

**Keywords:** air quality assessment, preference ranking organization methods for enrichment evaluations, Yangtze River Delta, air pollutant

## Abstract

A series of problems that are related to population, resources, environment, and ecology have emerged in recent years with the advancement of industrialization and urbanization in China. Especially, air pollution has become a severe trouble that directly endangers the health of residents. Accordingly, it is a need to make the assessment of air quality among cities, so that corresponding measures can be taken. For this purpose, ten major cities are selected as the research objects in Yangtze River Delta. Additionally, this study gathers and processes the data of five main air pollutants PM_2.5_, PM_10_, SO_2_, O_3_, and NO_2_, respectively. Furthermore, the maximizing deviation method is used to obtain the respective weight of these pollutants and the preference ranking organization method for enrichment evaluations (PROMETHEE) is introduced into the assessment of air quality among ten cities. As a result, the ranking of air quality in Ningbo, Wenzhou, Shanghai, and Shaoxing was at the fore from 2014 to 2017. Meanwhile, the performance of Ningbo has always kept the top two and Shaoxing’s ranking has risen since 2015. In addition, the air quality of Changzhou, Suzhou and Hangzhou was at an average level in the past four years. Moreover, the performance of Nanjing, Wuxi, and Zhenjiang was terrible when compared to other cities. Some useful suggestions have been proposed to control air quality based on the ranking results.

## 1. Introduction

China’s economy has made remarkable achievements over the past 40 years, but it leads to the huge damage to natural resources and environment. Water pollution, soil pollution, and other environmental issue have exploded in recent years, which have posed a great threat to the health of residents [1,2,3,4]. Particularly, air pollution has been more serious beyond expectation and the haze is the mostly attributed causes for pneumonia. Besides, it often results in a wide range of regional complex air hazards due to the diffusivity of air pollution. For instance, air quality in the Yangtze River Delta and Pearl River Delta has sharply deteriorated since 2013. According to the Jiangsu provincial environmental bulletin in 2017, all of the cities of the province failed to meet environmental standards regarding air quality. The main air pollutants, such as PM_2.5_, PM_10_, O_3_, and NO_2_, are severely out of limits.

Critical air contamination not only affects economic development, but it also gives rise to the harm to people’s health. The Chinese government has promulgated and implemented a series of laws and regulations, and invested a large amount of funds in solving air pollution in order to provide a fine environment in light of the stern environmental and public’s strong desire for clean air [5]. However, the effect of air pollution control is not significant, and the current air quality is far from satisfactory. Based on the report of China’s environmental state, 70.7 percent of urban air quality exceeded the standard in 2017. Thus, environmental governance still needs further improvement to practically increase the air quality. As air pollution has become a hot topic, lots of studies focus on the analysis of air pollution [6,7,8,9,10,11,12,13,14]. Combined with the data from national environmental monitoring center, the air pollution in China was evaluated, and it was found that there were significant differences [15,16]. The impacts of industrial structure, energy use, urban greening, and traffic congestion on air quality were specifically studied [17,18]. Moreover, joint cooperation between regional and local government is essential in productively addressing air pollution [19,20]. Many researchers have made the assessment of air quality through various methods.

Some pollution control regulations are recommended since PM_2.5_ and PM_10_ are found to be the most and responsible for extremely serious pollution in western China [21,22]. Urban air quality of 86 cities throughout China from 2001 to 2011 was estimated with the air pollution index, being composed by key pollutants [23]. Synthetic evaluation model in evaluating air quality by aggregating the four pollutants proved to be feasible [24]. The spatial–temporal evolution of air quality was explored in the Yangtze River Delta based on the generalization principal component analysis and the gravity center model [25]. The effect of emission-reduction measures on PM_2.5_ concentrations was evaluated and it can be used for simulating processes of air pollution, which might forecast about air quality in advance [26]. Furthermore, the air quality index has often been introduced into the study of air quality in recent years [27,28,29]. Daily air pollution data from nine monitoring stations in Wuhan was used to calculate the air quality index, which found that the number of polluted days was decreasing from 2013 to 2017 [30]. A new efficiency evaluation model based on evidential reasoning (IER) model and the interval data envelopment analysis (IDEA) model is proposed to evaluate air pollution [31]. Temporal and spatial heterogeneity of air pollution is evaluated in Lanzhou, China, which can provide guidance regarding air quality policies [1]. It is a need to further analyze the problem of air quality given the significance of air quality and the pursuit of a better living environment. It can be learnt that PM_2.5_, PM_10_, SO_2_, O_3_, and NO_2_ are the main air pollutants, which is one of main previous studies that contribute to the present research. In fact, each pollutant has different health effects and safety concentration. Previous studies regarding the air pollution based on the perspective are much few, which gives us a motivation of present research.

These pollutants have different concentrations in different areas. The multi-attribute decision-making (MADM) technique can be used to make an evaluation, s different air pollutants are involved in different areas. Multi-attribute utility theories [32] have indicated that many methods have been developed to address multi-criteria problems, AHP method, VIKOR method [33], TODIM method [34], ELECTRE method [35], MACBETH methods, etc. Different methods have different features. For AHP, it is difficult to reflect index interactions and the collection of data lies on experts’ experience. The complex computational process of VIKOR is very complex when handling terse data and it often fails to identify the weakness of alternatives. TODIM cannot handle compensation problem very well. Too many parameters are in the ELECTRE method. Preference Ranking Organization Method for Enrichment Evaluations (PROMETHEE) has distinct features when compared with these methods. The method can reflect many properties of attributes with less information loss [36]. The method can adopt lots of preference functions to evaluate alternatives that are based on the values of each criterion. It offers a novel idea about comparison among different alternatives that are difficult to distinguish. Besides, the PROMETHEE method has simplified the calculation procedure. Therefore, it is widely used in decision-making fields. A PROMETHEE-based preference ranking method is undertaken to evaluate a small set of motor vehicles based on constituents of their exhaust emissions [37]. Consolidated procedure employed PROMETHEE combined and Data Envelopment Analysis (DEA) are proposed to evaluate the environment in 30 European countries over the period 2008–2015, which highlights the relative strengths and flaws at the country-level for the considered pollutants [38]. PROMETHEE is used to rank the performance of operating copper smelters by systematically analyzing the technological, economic, and environmental parameters of the technologies applied [39]. The PROMETHEE V multi-criteria method is applied to evaluate and select from a variety of potentially feasible water resources development options in the Middle East [40]. PROMETHEE is developed to improve the robustness and practicality of decision results over traditional methods when selecting low-carbon building measures [41]. AHP-PROMETHEE is integrated to rank of municipal solid waste treatment alternatives, so that the analysis and decision making can be enhanced [42]. From the above analysis, it can be observed that PROMETHEE has been widely used in the pollutants control, energy development, and environment protection. The results have indicated that the approach is over traditional methods.

As one of the most important areas, the Yangtze River Delta plays an important part in the economic development of China. While considering the concern regarding air quality, ten major cities of the Yangtze River Delta are selected as research objectives, namely, Shanghai, Nanjing, Changzhou, Wuxi, Suzhou, Zhenjiang, Hangzhou, Ningbo, Wenzhou, and Shaoxing. It is a novel perspective for introducing PROMETHEE into the assessment of air quality. Subsequently, the ranking of each city can be obtained. By analyzing the ranking, some useful suggestions regarding how to control the air pollution can be proposed. These cities can follow these suggestions to improve air quality and realize better air quality.

## 2. Methodology

### 2.1. Data Sources

Referring to the study about air quality’s assessment [43,44,45,46,47], a basic data set of air quality, including SO_2_, NO_2_, PM_10_, PM_2.5_, and O_3_, are collected from urban statistical yearbook and environmental bulletin from 2014 to 2017 in order to assess the air quality. Ten cities are selected in this study respectively, Shanghai, Nanjing, Changzhou, Wuxi, Suzhou, Zhenjiang, Hangzhou, Ningbo, Wenzhou and Shaoxing. Taking Shanghai as an example, the data set obtained is as follows in Table 1.

### 2.2. Indices of Multi-Attribute Decision-Making (MADM)

The study of air quality is a multi-attribute decision-making problem, because it involved different air pollutants of respective cities. Therefore, the multi-attribute decision-making (MADM) technique can be adopted to make evaluation. It is necessary to figure out the types of indices to comprehensively evaluate the air quality of different cities.

In general, the indices in multi-attribute decision-making (MADM) problems can be classified into efficiency-typed index and cost-typed index. The bigger the attribute value, the better the efficiency-typed index, such as the labor productivity, product sales rate, energy conversion rate, and so on. The cost-typed index appears better when its attribute values varies from big to small, for example, air pollution index, governance cost belong to these indices. A conversion criterion was proposed to eliminate the influence caused by the difference of dimension and unit due to the different evaluation index having a different dimension and unit [48].

Assuming a MADM problem, the alternative set is $A=\left\{A_{1},A_{2},\ldots ,A_{m}\right\}$, the attribute set is $C=\left\{C_{1},C_{2},\ldots ,C_{n}\right\}$ and the evaluation value of alternative A with regard to attribute C is $x_{ij}\left(i=1,2,\ldots ,m;j=1,2,\ldots ,n\right)$, the value after eliminating the influence that is caused by the difference of dimension and unit is represented by $y_{ij}\left(i=1,2,\ldots ,m;j=1,2,\ldots ,n\right)$. The conversion criterion of efficiency-typed index is defined, as follows:

(1) $$y_{ij}=\frac{x_{ij}-x_{j}^{min}}{x_{j}^{max}-x_{j}^{min}},\;\;\;\;\left(i=1,2,\ldots ,m;j=1,2,\ldots ,n\right)$$

where $x_{j}^{max}$ and $x_{j}^{min}$ represent the maximum and the minimum of the value of attribute $C_{j}$, respectively. The bigger the value of $x_{ij}$, the bigger the $y_{ij}$ and the higher ranking of $A_{i}$ under $C_{j}$. Besides, the priority of the alternatives will not change along with the converted values.

The conversion criterion of cost-typed index is defined, as follows:

(2) $$y_{ij}=\frac{x_{j}^{max}-x_{ij}}{x_{j}^{max}-x_{j}^{min}},\;\;\;\;\left(i=1,2,\ldots ,m;j=1,2,\ldots ,n\right)$$

where $x_{j}^{max}$ and $x_{j}^{min}$ represent the maximum and the minimum of the value of attribute $C_{j}$, respectively. The smaller the value of $x_{ij}$, the bigger the $y_{ij}$ and the higher ranking of $A_{i}$ under $C_{j}$. The priority of the alternatives will not change.

The World Health Organization (WHO) Air Quality Guidelines (AQG) launched in 2006 is used as a reference to express the different health effect of each air pollutant [49]. For the convenience of further research, the evaluation value of each air pollutant assessed in the empirical research is obtained by subtracting the AQG from initial pollution value. If the initial value of air pollutant is lower than AQG, then we let it equal to 0. For example, in Table 2, the 8 h concentration of O_3_ in Shanghai is 149 μg/m^3^ and the AQG is 100 μg/m^3^, thus the evaluation value of O_3_ is 40; the 8 h concentration of O_3_ in Nanjing is 57 μg/m^3^, so the evaluation value of O_3_ is 0.

Based on the conversion criterion mentioned above, the efficiency-typed index and cost-typed index can both be compared in the same dimension and unit. In order to further explain the conversion criterion, an example will be given in the following. Table 3 shows a data set of SO_2_ and O_3_ related to ten cities in 2014. As can be seen in the table, air pollution index is a kind of cost-typed index and Equation (2) is used to calculate the results. For example, Changzhou has the highest content of SO_2_, while Shanghai, Ningbo and Wenzhou have the lowest, so the conversion value of Shanghai, Ningbo, and Wenzhou is 1 and the conversion value of each remained city is smaller, which means that Shanghai, Ningbo, and Wenzhou rank the highest and Changzhou ranks the lowest. As a result, $x_{j}^{max}-x_{j}^{min}=16-0=16$. Table 3 shows all of the examples.

### 2.3. Preference Ranking Organization Method for Enrichment Evaluations (PROMETHEE)

The PROMETHEE method employs the outranking principles to rank the alternatives, combined with ease of use and decreased complexity. The method uses the preference function $P_{j}\left(a_{i},a_{k}\right)$, which is a function of the distance $d_{j}$ between alternative $a_{i}$ and $a_{k}$ regarding criterion *j.* Six basic types are proposed in order to facilitate the selection of a specific preference function, as follows [50].

(1) General criterion

(3) $$P_{j}\left(a_{i},a_{k}\right)=\{\begin{array}{ll} 0, & d_{j}\left(a_{i},a_{k}\right)\leq 0\\ 1, & d_{j}\left(a_{i},a_{k}\right)>0 \end{array}$$

(2) U-shaped criterion

(4) $$P_{j}\left(a_{i},a_{k}\right)=\{\begin{array}{ll} 0, & d_{j}\left(a_{i},a_{k}\right)\leq \upsilon \\ 1, & d_{j}\left(a_{i},a_{k}\right)>\upsilon \end{array}$$

(3) Linear criterion

(5) $$P_{j}\left(a_{i},a_{k}\right)=\{\begin{array}{cc} 0, & d_{j}\left(a_{i},a_{k}\right)\leq 0\\ \frac{d_{j}\left(a_{i},a_{k}\right)}{\upsilon }, & 0<d_{j}\left(a_{i},a_{k}\right)\leq \upsilon \\ 1, & d_{j}\left(a_{i},a_{k}\right)>0 \end{array}$$

(4) Multi-level criterion

(6) $$P_{j}\left(a_{i},a_{k}\right)=\{\begin{array}{ll} 0, & d_{j}\left(a_{i},a_{k}\right)\leq \upsilon \\ \frac{1}{2}, & \upsilon <d_{j}\left(a_{i},a_{k}\right)\leq \omega \\ 1, & d_{j}\left(a_{i},a_{k}\right)>\omega \end{array}$$

(5) Linear indifference interval criterion

(7) $$P_{j}\left(a_{i},a_{k}\right)=\{\begin{array}{cc} 0, & d_{j}\left(a_{i},a_{k}\right)\leq \upsilon \\ \frac{d_{j}\left(a_{i},a_{k}\right)-\omega }{\omega -\upsilon }, & \upsilon <d_{j}\left(a_{i},a_{k}\right)\leq \omega \\ 1, & d_{j}\left(a_{i},a_{k}\right)>\omega \end{array}$$

(6) Gaussian criterion

(8) $$P_{j}\left(a_{i},a_{k}\right)=\{\begin{array}{cc} 0, & d_{j}\left(a_{i},a_{k}\right)\leq 0\\ 1-e^{-\;\frac{d_{j}^{2}\left(a_{i},a_{k}\right)}{2\sigma^{2}}}, & d_{j}\left(a_{i},a_{k}\right)>0 \end{array}$$

No matter which type of the preference function is selected, $d\left(a_{i},a_{k}\right)=f\left(a_{i}\right)-f\left(a_{k}\right)$ should be calculated first, where $f\left(a_{i}\right)$ and $f\left(a_{k}\right)$ are the evaluation of two alternatives on criterion *j*.

The values of the multi-attribute preference index *H* can be determined according to the weight of the attributes after obtaining the preference values between alternatives. The closer the value of *H* is to one, the better the superiority of the alternative $a_{i}$.

(9) $$H\left(L_{i}\left(p\right),L_{k}\left(p\right)\right)=\sum_{j=1}^{n}\omega_{j}\times P_{j}\left(L_{i}\left(p\right),L_{k}\left(p\right)\right)$$

The preference index is used to compute the leaving flow:

(10) $$\varphi^{+}\left(L_{i}\left(p\right)\right)=\sum_{k=1}^{m}H\left(L_{i}\left(p\right),L_{k}\left(p\right)\right)=\sum_{j=1}^{n}\omega_{j}\times P_{j}\left(L_{i}\left(p\right),L_{k}\left(p\right)\right)$$

Entering flow:

(11) $$\varphi^{-}\left(L_{i}\left(p\right)\right)=\sum_{k=1}^{m}H\left(L_{k}\left(p\right),L_{i}\left(p\right)\right)=\sum_{j=1}^{n}\omega_{j}\times P_{j}\left(L_{k}\left(p\right),L_{i}\left(p\right)\right)$$

And net flow:

(12) $$\varphi \left(L_{i}\left(p\right)\right)=\varphi^{+}\left(L_{i}\left(p\right)\right)-\varphi^{-}\left(L_{i}\left(p\right)\right)$$

The leaving flow represents the dominant position of an alternative relative to other alternatives. It is a measure of the outranking character. The entering flow is a measure of an outranked character. The net flow can be obtained by leaving flow minus entering flow. Finally, the larger the value of net flow, the higher the ranking of the alternative [51].

### 2.4. Weight of Attributes

When considering that the variation of attribute weights might influence the final ranking of alternatives [52], it is necessary to develop a new method for determining attribute weights based on the maximizing deviation method [53].

Firstly, a multi-objective program is constructed based on the decision-making matrix G. Using $W_{ij}$ to represent the sum priority of $A_{i}$ in attribute $C_{j}$.

(13) $$W_{ij}=\sum_{k=1,k\neq i}^{m}\omega_{j}P\left(A_{ij}\geq A_{kj}\right)\;\;\;\;\;\left(i=1,2,\ldots ,m;j=1,2,\ldots ,n\right)$$

where the value of $P\left(A_{ij}\geq A_{kj}\right)$ can be obtained by the preference function:

(14) $$W_{j}=\sum_{i=1}^{m}W_{ij}=\sum_{i=1}^{m}\sum_{k=1,k\neq i}^{m}\omega_{j}P\left(A_{ij}\geq A_{kj}\right)\;\;\;\;\;\left(j=1,2,\ldots ,n\right)$$

According to the maximizing deviation method [48], if an attribute makes little difference on assessment values among alternatives, the attribute should be assigned a smaller weight. While an attribute makes great difference on assessment values among alternatives, the attribute should be assigned a larger weight. Subsequently, the multi-objective program is constructed as:

(15) $$\begin{array}{c} max\;W_{j}=\sum_{i=1}^{m}\sum_{k=1,k\neq i}^{m}\omega_{j}P\left(A_{ij}\geq A_{kj}\right)\;\;\;\;\;\left(j=1,2,\ldots ,n\right)\\ \;s.t\{\begin{array}{l} \sum_{j=1}^{n}\omega_{j}=1,\\ \omega_{j}\geq 0,\;\;\;j=1,2,\ldots ,n. \end{array} \end{array}$$

Let $\theta =min\left\{W_{1},W_{2},\ldots ,W_{n}\right\}$, then Equation (15) is transformed into a single objective program, as:

(16) $$\begin{array}{c} max\;\theta \\ \;s.t\{\begin{array}{l} \sum_{i=1}^{m}\sum_{l=1,l\neq i}^{m}\omega_{j}P\left(L_{ij}\left(p\right)\geq L_{lj}\left(p\right)\right)\geq \theta \;\;\left(j=1,2,\ldots ,n\right)\\ \sum_{j=1}^{n}\omega_{j}=1,\\ \omega_{j}\geq 0,\;\;\;j=1,2,\ldots ,n. \end{array} \end{array}$$

For convenience, set $C_{j}=\sum_{i=1}^{m}\sum_{l=1,l\neq i}^{m}P\left(L_{ij}\left(p\right)\geq L_{lj}\left(p\right)\right)$ in the following.

It is easy to see that Equation (16) is equivalent to Equation (17), as follows:

(17) $$\begin{array}{c} max\;\theta \\ \;s.t\{\begin{array}{l} \sum_{i=1}^{m}\sum_{l=1,l\neq i}^{m}\omega_{j}P\left(L_{ij}\left(p\right)\geq L_{lj}\left(p\right)\right)\geq \theta \;\;\left(j=1,2,\ldots ,n\right)\\ \sum_{j=1}^{n}\omega_{j}=1,\\ \omega_{j}\geq 0,\;\;\;j=1,2,\ldots ,n. \end{array} \end{array}$$

Here, we can adopt the Lagrange function to solve the model:

(18) $$L\left(\theta ,\lambda_{j},\mu ,\omega_{j}\right)=\theta +\sum_{j=1}^{n}\lambda_{j}\left(\omega_{j}C_{j}-\theta \right)+\mu \left(\sum_{j=1}^{n}\omega_{j}-1\right)$$

Setting the partial derivations to be zero:

(19) $$\begin{array}{ll} \frac{\partial }{\partial \theta }L\left(\theta ,\lambda_{j},\mu ,\omega_{j}\right)=1-\sum_{j=1}^{n}\lambda_{j}=0 & \frac{\partial }{\partial \mu }L\left(\theta ,\lambda_{j},\mu ,\omega_{j}\right)=\sum_{j=1}^{n}\omega_{j}-1=0\\ \frac{\partial }{\partial \lambda_{j}}L\left(\theta ,\lambda_{j},\mu ,\omega_{j}\right)=\omega_{j}C_{j}-\theta =0 & \frac{\partial }{\partial \omega_{j}}L\left(\theta ,\lambda_{j},\mu ,\omega_{j}\right)=\lambda_{j}C_{j}+\mu =0 \end{array}$$

Thus, $\omega_{j}C_{j}=\theta$, $\lambda_{j}C_{j}=-\mu$, $\sum_{j=1}^{n}\lambda_{j}=1$, $\sum_{j=1}^{n}\omega_{j}=1$. It follows that $\sum_{j=1}^{n}\omega_{j}=\sum_{j=1}^{n}\theta /C_{j}=1$. Hence, the result is $\theta =1/\sum_{j=1}^{n}\frac{1}{C_{j}}$.

According to the first and second constraints of Equation (17), one has $\sum_{j=1}^{n}\theta /C_{j}\leq \sum_{j=1}^{n}\omega_{j}=1$, therefore, $\theta \leq 1/\sum_{j=1}^{n}\frac{1}{C_{j}}$. Hence, $\theta =1/\sum_{j=1}^{n}\frac{1}{C_{j}}$ is the maximum of θ. Meanwhile, the optimal solution of the single objective program is:

(20) $$\omega_{j}{}^{*}=\frac{\theta }{C_{j}}=\frac{1}{C_{j}\sum_{j=1}^{n}\frac{1}{C_{j}}}\;\;\;\left(j=1,2,\ldots ,n\right)$$

The maximizing deviation method can be implemented in the problem, where the weight of attributes is completely unknown [48]. This method can expand the differences between attributes and make the ranking order clearer and easier to understand; it can also improve the rationality and validity of the results.

### 2.5. Evaluate Procedure

Based on above discussion, the procedure regarding how to evaluate air pollution is proposed, as follows:

**Step 1:** Define a multi-attribute decision-making problem and the air pollutants set (SO_2_, NO_2_, PM_10_, PM_2.5_, and O_3_) of that is $C=\left\{C_{1},C_{2},\ldots ,C_{n}\right\}$, the set of alternatives (ten cities) is $A=\left\{A_{1},A_{2},\ldots ,A_{m}\right\}$, the value of A with regard to C is $x_{ij}\left(i=1,2,\ldots ,n;j=1,2,\ldots ,m\right)$, the generated multi-attribute decision-making matrix is G.

**Step 2:** Normalize the evaluation value by subtracting the AQG from initial pollution value and the conversion approach is adopted to eliminate the influence that is caused by the difference of dimension and unit.

**Step 3:** Calculate the distance value $d=f\left(A_{i}\right)-f\left(A_{k}\right)$ between different alternatives, the bigger the value, the larger the differences between $A_{i}$ and $A_{k}$.

(21) $$d\left(A_{ij},A_{kj}\right)=\{\begin{array}{cc} y_{i}-y_{k} & y_{ij}>y_{kj}\\ 0 & y_{ij}\leq y_{kj} \end{array}$$

**Step 4:** Gaussian criterion preference function is used to obtain the preference degree $P_{ij}\left(A_{ij},A_{kj}\right)$. When the discrepancy between $f\left(A_{i}\right)$ and $f\left(A_{k}\right)$ is less than or equal to 0, it indicates that the alternative $A_{i}$ and $A_{k}$ are indifferent. When the discrepancy between them is greater than 0, it denotes that the alternative $A_{i}$ is strictly better than $A_{k}$.

(22) $$P_{ij}\left(A_{ij},A_{kj}\right)=1-e^{-\;\frac{d^{2}\left(A_{ij},A_{kj}\right)}{2\sigma^{2}}}$$

**Step 5:** The weight of air pollutants can be calculated while using the method of maximizing deviation. Construct a multi-objective program that is based on the preference degree $P_{ij}\left(A_{ij},A_{kj}\right)$ and convert it to the single objective program (17). Solving the program and generating the value of $\omega_{j}{}^{*}$.

**Step 6:** Calculate the preference index $H\left(A_{i},A_{k}\right)$ based on Equation (9).

**Step 7:** Derive the leaving flow $\varphi^{+}$, entering flow $\varphi^{-}$, and net flow $\varphi \left(A_{i}\right)$ by Equations (10) and (11). Subsequently, rank the alternatives and analyze the results, finally put the policy suggestions based on the analysis above forward.

**Step 8:** End.

## 3. Case Study

This paper takes ten major cities in the Yangtze River Delta as the research object, namely, Shanghai, Nanjing, Changzhou, Wuxi, Suzhou, Zhenjiang, Hangzhou, Ningbo, Wenzhou, and Shaoxing. Based on the air pollutant data that were collected, the method of multi-attribute decision-making is applied into the case study to gain the ranking of air quality among ten cities. The detailed process is as follows:

**Step 1:** Define a multi-attribute decision-making problem and the attribute set: the content of SO_2_ (C1), NO_2_ (C2), PM_10_ (C3), PM_2.5_ (C4), O_3_ (C5), the set of alternatives: Shanghai (A1), Nanjing (A2), Changzhou (A3), Wuxi (A4), Suzhou (A5), Zhenjiang (A6), Hangzhou (A7), Ningbo (A8), Wenzhou (A9), and Shaoxing (A10). $x_{ij}\left(i=1,2,\ldots ,10;j=1,2,\ldots ,5\right)$ represents the value of air pollutants of cities from 2014 to 2017 and generate the decision-making matrix G.

**Step 2:** Normalize the evaluation value by subtracting the AQG from initial pollution value and eliminate the influences that are caused by the difference of dimension and unit. We find that the lower the value, the better the air pollution index, so the air pollution index belongs to the cost-typed index. The conversion value can be calculated by Equation (2) and is shown in Table 4.

**Step 3:** After the conversion process, the distance value $d\left(A_{ij},A_{kj}\right)$ can be obtained based on Equation (21).

**Step 4:** Based on the Gaussian criterion preference function of Equation (22), the preference degree $P_{ij}\left(A_{ij},A_{kj}\right)$ can be computed by the distance value $d\left(A_{ij},A_{kj}\right)$. DMs select the parameter according to the actual needs in the decision process and its subjective preferences (σ=1).

**Step 5:** Using the maximizing deviation method to calculate the weight of attributes. The weights can characterize the significance of each attribute in the MADM by using the maximizing deviation method. If an attribute makes little difference on assessment values among alternatives, the attribute should be assigned a smaller weight. The attribute should be assigned a larger weight while an attribute makes great difference on assessment values among the alternatives. Table 5 shows the results.

**Step 6:** Calculate the preference index $H\left(A_{i},A_{k}\right)$ by using the weighted method. Table 6 shows the results in 2014 based on the preference degree $P_{ij}\left(A_{ij},A_{kj}\right)$.

**Step 7:** Derive the leaving flow $\varphi^{+}$, entering flow $\varphi^{-}$, and net flow $\varphi \left(A_{i}\right)$ by Equations (10) and (11). Table 7 shows the results.

**Figure 1:**
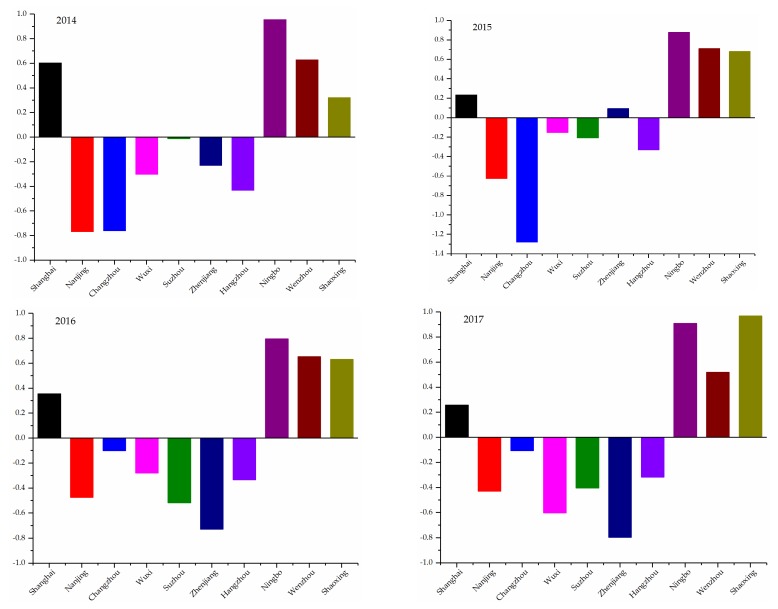
The net flow of eleven regions from 2014 to 2017.

To give the annual presentation clearer, Figure 1 is used to graphically illustrate the ranking order of ten cities. It is obvious that value of net flow fluctuates around 0. During the evaluation procedure above, the value of net flow is obtained by subtracting entering flow from leaving flow. Entering flow represents the priority over other alternatives, the larger the value, the better the superiority. Leaving flow indicates the dominance of an alternative over other alternatives, the smaller the value, the better the superiority. Thus, the larger the value of net flow, the higher the ranking order.

Figure 1 is used to develop a visualized and easy way for readers to comprehend. It is distinctly that the place of Ningbo Wenzhou, Shanghai, and Shaoxing are always ranking in the top four and the distribution of four histograms is similar, which means that the ranking order of cities is stable in four years.

## 4. Results and Discussion

### 4.1. Results

**Figure 2:**
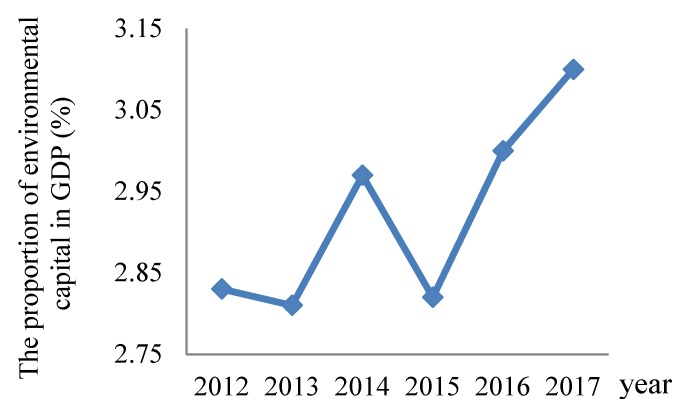
The change of proportion of environmental governance capital in GDP of Shanghai.

Shanghai ranked forefront among ten cities during the past five years, which indicated that the environmental governance has made great achievements. The investment on environmental protection in Shanghai has gradually increased since 2013, and its proportion of environmental governance capital in GDP has already reached 3.1%, which provides good financial support for the further implementation of air pollution control. Figure 2 presents the change of proportion of environmental governance capital in GDP of Shanghai. As the most developed region in Yangtze River Delta, Shanghai’s environmental protection has an important guiding role for other cities.

On the contrary, the performance of Nanjing was far from satisfactory when compared with Shanghai. It ranked relatively later in the first two years among ten chosen cities. Additionally, Nanjing was behind in the ranking in terms of air quality in the remaining years. As a traditional industrial base in China, it has been in an unbalanced state of the development of heavy and light industries that leads to a high proportion of traditional heavy industry. Particularly, the four industries, including power, steel, petrochemical, and cement, account for approximately 95% of the total industrial energy consumption, which result in the awful air quality.

In general, Wuxi’s air quality has decreased a lot as compared with the first two years. There existed a significant fluctuation from 2014 to 2017, where the ranking of it fell from sixth to ninth rapidly. Meanwhile, ninety percent of comprehensive energy consumption in Wuxi is occupied by heavy industry that has brought great difficulties to environmental governance.

Moreover, Changzhou’s best place on air quality was sixth in past four years, while it has been at the bottom for other years. With the progress of construction of ecological civilization and environmental protection, industrial pollution control was basically completed and the mission of total air pollutant emission reduction was fulfilled in 2017. As a result, the air quality of Changzhou in 2016 and 2017 increased by three places as compared with two years before.

Contiguous to Wuxi, Suzhou has been ranked behind fifth for three times and the worst place was the penultimate from 2014 to 2017. Suzhou has increased commitment to environmental protection in the following years after ranking ninth among ten selected cities in terms of air quality. Especially, 956 air pollution control projects were completed in 2017, bringing it back to seventh.

The overall performance of Zhenjiang’s air quality was poor in the latter two years. Although Zhenjiang’s environmental protection has been advancing since 2012, it has not been capable of improving air quality. Many chemical plants are clustered in Zhenjiang. On one hand, they contribute to the development of economy. On the other hand, the toxic gases that are emitted by the factories do great harm to the body of people. It is a great challenge for local government to balance development and environment. Hence, the improvement of air quality in Zhenjiang needs a long and continuous process.

As the capital of Zhejiang province, Hangzhou’s air quality was at an average level generally. It ranked seventh or eighth in four years. The implementation plan for air pollution prevention and control and related rules were promulgated and put into effect in 2017. Meanwhile, Hangzhou has launched an intensive campaign to control air pollution and made 233 enterprises upgrade the waste disposal equipment, which reduced 9974 tons of volatile organic compounds. Accordingly, the air quality has become better.

Furthermore, when considering the air quality of Ningbo, it had remarkable performance as compared with other cities, which occupied the top two for four years in a row. Additionally, Ningbo has always insisted on the treatment of pollution and environmental protection. Over the past five years, a great deal of fund has been invested to cut the emission of air pollutants. At present, the management of volatile organic compounds in Ningbo has reached the domestic leading level.

In addition, Wenzhou ranked at the forefront for air quality of selected objects from 2014 to 2017. The performance of air pollution control of various district governments in Wenzhou is strictly supervised and evaluated, which ensures the effective implementation of environmental governance. Besides, the establishment of joint consultation mechanism on air quality plays an important role in the reduction of atmospheric pollutants in Wenzhou. Moreover, the air quality in Shaoxing has been gradually improved and the ranking has remained first for two consecutive years since 2016. Local government focuses on the environmental governance and it adjusts the structure of energy consumption based on the regulations on the prevention and control of air pollution of Shaoxing, which significantly reduces the air pollutant emission.

### 4.2. Compare with the Air Quality Indexes

In fact, there are a lot of criteria for judging air pollution quality. There are some differences among different standards. Air quality index is widely adopted among these standards. The latest air quality index consists of concentration values of ozone (O_3_), fine suspended particulate matter (PM_2.5_), suspended particulate matter (PM_10_), carbon monoxide (CO), sulfur dioxide (SO_2_), and nitrogen dioxide (NO_2_). According to the influence of the human health, the individualized air quality index (*IAQI*) was computed, as follows:

(23) $$IAQI_{p}=\frac{IAQI_{Hi}-IAQI_{Lo}}{BP_{Hi}-BP_{Lo}}\left(C_{p}-BP_{Lo}\right)+IAQI_{Lo}$$

in which $IAQI_{p}$ is *IAQI* of pollutant item *P*, $C_{p}$ is the concentration value of pollutant item *P*, $BP_{Lo}$ is the lower limit for classification of pollutant and CPs, $IAQI_{Hi}$ is the upper limit of *AQI* classification corresponding to $BP_{Hi}$ for pollutant items, and $IAQI_{Lo}$ is the lower grading limit of *AQI* value corresponding to $BP_{Lo}$ for pollutant items.

Then, the maximum of *IAQI* is selected as the final index as *AQI*, as follows:

(24) $$AQI=\max\left\{IAQI_{1},IAQI_{2},\ldots ,IAQI_{n}\right\},n=1,\;2,\ldots ,\;6$$

From the calculation process of *AQI*, it can be noticed that *AQI* is different from our proposed method. The maximum of *IAQI* is considered as the *AQI*. It indicates that the weight of the maximum of *IAQI* is one and other weights of pollutants are zero. Besides, the range of *AQI* is between 0 and 500. If *IAQI* is bigger than 500, then *AQI* has to be 500. It is just used to calculate the *AQI* of each city.

However, the method and aim of the paper are different from *AQI*. Firstly, we want to make differences among these cities more distinct. The maximizing deviation method is used to calculate the weight. Second, each evaluation results have no upper limit. Third, the main purpose is to find useful approaches to deal with air pollution [54].

### 4.3. Discussion

The Yangtze River Delta is one of the most important areas in China’s economic development. It has an important strategic position in China’s future modernization. This paper evaluates the air quality in ten major cities of Yangtze River Delta. Some useful policy suggestions are put forward based on the research above in order to realize the sustainable development of urban. They will have a positive significance for improvement of urban air quality. The suggestions are proposed, as follow:

(1) Improve joint mechanisms for air pollution control between regions.

Air pollution has the characteristics of transmission, and the regional transmission pollution is becoming more and more obvious recently, due to the “space spillover” effect. At present, the fragmentation of the air pollution control in China is territorial governance model, which cannot effectively deal with air pollution. However, the joint mechanisms for air pollution control between regions can solve it. When considering the different emission standards and regulations in various regions, coordinated management cannot be fundamentally resolved only through direct consultation in each region. Therefore, unifying the rules, institutions, and standards in different regions from a legal perspective, the administrative barriers can be broken and the requirements for inter-regional air defense can be implemented accordingly. For example, establish a regional joint leadership group to strengthen the coordination of inter-regional work; establish a joint conference mechanism for the prevention and control of air pollution. When considering that the trend of air pollution in 11 regions from 2014 to 2017 did not change significantly based on our study, the Yangtze River Delta Regional Environmental Meteorology Integration Platform was set up in 2018 for the purpose of improving joint mechanisms. Focus on three aspects: precise control of atmospheric pollution, forecasting of heavily polluted weather, and long-term regulation of environment. Joint mechanisms for information sharing and transmission between the meteorological and environmental protection departments will be further developed in the Yangtze River Delta.

(2) Improve laws and regulations related to air pollution

Improve the emergency response systems and raising the threshold for emission permits. The government should promote the revision of air pollution prevention laws and increase the penalties for illegal enterprises that cause major pollution to the environment. Meanwhile, they need to amend new laws on environmental protection and motor vehicle pollution prevention to regulate the environmental public interest litigation system. In addition, each region can formulate local emission standards, oil standards, automobile fuel consumption standards, and heating metering standards, etc., according to their practical situation. Gradually improve the evaluation index system for pollution prevention technology and cleaner production capacity in related industries. Every year, the government will promulgate “Plan for Comprehensive Control of Air Pollution in the Yangtze River Delta” to tackle environmental pollution in autumn and winter. Increase the penalties for companies that fail to meet the pollution standards and use laws to ensure air quality.

(3) Optimize and upgrade the industrial structure

When compared with the Pearl River Delta, another economic center of China, the proportion of heavy industries in the Yangtze River Delta is relatively large. The development of industrialization has caused atmospheric pollution and made contribution to the increase of inhalable particulates in the atmosphere. It is essential to change the way of economic development and follow the path of sustainable development. The most important thing in transforming the development model is to optimize the industrial structure and increase the intensity of the structural reforms. For example, we can reduce the proportion of the secondary industry and develop the industry about environmental protection and biological manufacturing; increase the proportion of the tertiary industry or accelerate the development and optimization of modern service industry; encourage enterprises to adopt advanced production technology. Less air pollutants will be emitted by these efforts.

(4) Strengthen the control of automobile exhaust and dust control

The number of vehicles is also increasing with the increase of population density and the improvement of living standards, and the exhaust pollution is becoming increasingly serious, especially in developed areas. The car parc of per one hundred people in the Yangtze River Delta is high throughout the country. The exhaust gas from automobile exhaust contains hundreds of different compounds, the pollutants include solid suspended particulates, CO, CO_2_, hydrocarbons, nitrogen oxides, lead, and sulfur oxides. It significantly contributes to the inhalable particles of the main pollutants in the atmosphere; the air pollutants studied in this paper mainly comes from automobile exhaust. It is necessary to formulate standards that regulate automobile emissions in order to control exhaust and restrain the rapid increase of automobiles. Develop public transport facilities and encourage residents to drive fewer cars. Give appropriate discounts to make residents aware of the convenience, economy, and environmental protection of public transportation.

(5) Increase the green area of the city

The increase in green area can improve air quality, because green plants can absorb pollutants, such as SO_2_ and dust in the air, and achieve the purpose of purifying the air quality. Previous studies have shown that the per capita park green space is highly correlated with PM_10_. The increase in per capita park area can control the content of air pollutants and reduce PM_10_ in the air. Therefore, increasing the construction of urban green belts can improve the air quality.

(6) Increase public participation in protecting the environment

The prevention and control of air pollution is not only the responsibility of the government, but also the participation of the entire public in environmental protection and supervision. Government departments should help more residents to establish awareness of protecting the atmosphere, and individuals should more consciously protect air quality. The environmental protection department should ensure that information, such as inspection and punishment of enterprises, is fully disclosed, so that citizens can oversee government departments publicly. All of the citizens must take the initiative to understand common sense of nature and establish ecological awareness.

## 5. Conclusions

This study aims to evaluate the air quality in major cities of Yangtze River Delta. For this purpose, ten cities of the Yangtze River Delta are selected as research objectives, namely, Shanghai, Nanjing, Changzhou, Wuxi, Suzhou, Zhenjiang, Hangzhou, Ningbo, Wenzhou, and Shaoxing. PROMETHEE is used to study the air quality of chosen cities from 2014 to 2017. The PROMETHEE method employs the outranking principles to rank the alternatives combined with ease of use and decreased complexity, and the maximizing deviation method can avoid the situation that individual extreme values affect the overall distribution as much as possible. The synthetically method proposed above can help to improve the accuracy of decision.

It is found that Shanghai, Ningbo, Wenzhou, and Shaoxing ranked forefront among ten cities during the past five years. Especially, Ningbo always came first and second, Shaoxing’s ranking kept going up until it became first. Furthermore, the performance of air quality in Changzhou, Suzhou, and Hangzhou was at a middle level during in the observation period. Meanwhile, the air quality in Nanjing, Wuxi, and Zhenjiang was awful as compared to other cities. Particularly, due to the high proportion of heavy industry in the structure of economy, it poses enormous challenges to environmental governance. Therefore, some useful policy recommendations are provided.

## Figures and Tables

**Table 1:** Air pollutants and their annual average concentration in Shanghai from 2014 to 2017.

Pollutant	Unit	Annual Average Concentration			
		2014	2015	2016	2017
SO_2_	μg/m^3^	18	17	15	12
NO_2_	μg/m^3^	45	46	43	44
PM_10_	μg/m^3^	71	69	59	55
PM_2.5_	μg/m^3^	52	53	45	39
8h O_3_	μg/m^3^	149	160.94	164	181

**Table 2:** Air Quality Guidelines (AQG) launched by WHO in 2006.

Air Pollutants	Mean Concentration	AQG
SO_2_	Annual	20 μg/m^3^
	10 min	500 μg/m^3^
NO_2_	Annual	40 μg/m^3^
	One hour	200 μg/m^3^
PM_10_	Annual	20 μg/m^3^
	24 h	50 μg/m^3^
PM_2.5_	Annual	10 μg/m^3^
	24 h	25 μg/m^3^
O_3_	8 h	100 μg/m^3^

**Table 3:** The conversion value of air pollution index.

City	SO_2_ μg/m^3^	Evaluation Value	Converted Value	O_3_ μg/m^3^	Evaluation Value	Converted Value
Shanghai	18	0	1	149	49	0.3099
Nanjing	25	5	0.6875	57	0	1
Changzhou	36	16	0	171	71	0
Wuxi	34	14	0.125	100	0	1
Suzhou	24	4	0.75	95	0	1
Zhenjiang	24	4	0.75	132.5	32.5	0.5423
Hangzhou	21	1	0.9375	170	70	0.0141
Ningbo	17	0	1	143.4	43.4	0.3887
Wenzhou	17	0	1	134	34	0.5211
Shaoxing	29	9	0.4375	93	0	1

**Table 4:** The conversion value of index.

**City**	**2014**					**2015**				
	**SO_2_**	**NO_2_**	**PM_10_**	**PM_2.5_**	**O_3_**	**SO_2_**	**NO_2_**	**PM_10_**	**PM_2.5_**	**O_3_**
Shanghai	1.000	0.643	1.000	0.784	0.310	1.000	0.571	1.000	0.471	0.142
Nanjing	0.688	0.000	0.000	0.000	1.000	1.000	0.286	0.182	0.235	0.000
Changzhou	0.000	1.000	0.365	0.245	0.000	0.000	0.643	0.000	0.118	0.085
Wuxi	0.125	0.643	0.423	0.209	1.000	0.400	0.929	0.273	0.000	1.000
Suzhou	0.750	0.071	0.712	0.281	1.000	0.900	0.000	0.667	0.176	1.000
Zhenjiang	0.750	0.571	0.308	0.209	0.542	0.500	0.857	0.606	0.118	0.915
Hangzhou	0.938	0.286	0.481	0.331	0.014	1.000	0.357	0.515	0.235	0.056
Ningbo	1.000	0.929	0.962	1.000	0.389	1.000	0.786	1.000	0.941	0.507
Wenzhou	1.000	0.286	0.923	1.000	0.521	1.000	0.643	0.909	1.000	0.324
Shaoxing	0.438	1.000	0.577	0.388	1.000	0.900	1.000	0.697	0.471	1.000
	**2015**					**2016**				
	**SO_2_**	**NO_2_**	**PM_10_**	**PM_2.5_**	**O_3_**	**SO_2_**	**NO_2_**	**PM_10_**	**PM_2.5_**	**O_3_**
Shanghai	1.000	0.727	1.000	0.533	0.238	1.000	0.500	1.000	0.895	0.036
Nanjing	1.000	0.609	0.000	0.340	0.000	1.000	0.125	0.400	0.842	0.060
Changzhou	1.000	1.000	0.160	0.267	0.297	1.000	0.875	0.486	0.474	0.167
Wuxi	1.000	0.364	0.122	0.000	0.976	1.000	0.250	0.314	0.579	0.000
Suzhou	1.000	0.000	0.504	0.467	0.202	1.000	0.000	0.686	0.684	0.131
Zhenjiang	0.000	1.000	0.198	0.200	1.000	1.000	0.625	0.000	0.000	0.905
Hangzhou	1.000	0.545	0.237	0.280	0.155	1.000	0.375	0.514	0.579	0.131
Ningbo	1.000	1.000	0.885	0.933	0.512	1.000	1.000	1.000	1.000	0.536
Wenzhou	1.000	0.909	0.618	1.000	0.512	1.000	0.875	0.714	0.947	0.464
Shaoxing	1.000	1.000	0.656	0.533	1.000	1.000	1.000	0.771	0.789	1.000

**Table 5:** Attribute weights.

City	2014	2015	2016	2017
C1	0.1967	0.2076	0.2462	0.0000
C2	0.1899	0.2448	0.1939	0.2017
C3	0.2422	0.2049	0.1845	0.2780
C4	0.2084	0.2069	0.2198	0.3131
C5	0.1628	0.1359	0.1556	0.2073

**Table 6:** Preference index *H* of cities in 2014.

**A1**		**A2**		**A3**		**A4**	
H(A1,A2)	0.1953	H(A2,A1)	0.0345	H(A3,A1)	0.0117	H(A4,A1)	0.0345
H(A1,A3)	0.1574	H(A2,A3)	0.1054	H(A3,A2)	0.0965	H(A4,A2)	0.0607
H(A1,A4)	0.1315	H(A2,A4)	0.0288	H(A3,A4)	0.0119	H(A4,A3)	0.0660
H(A1,A5)	0.0693	H(A2,A5)	0.0000	H(A3,A5)	0.0665	H(A4,A5)	0.0286
H(A1,A6)	0.0900	H(A2,A6)	0.0162	H(A3,A6)	0.0172	H(A4,A6)	0.0183
H(A1,A7)	0.0700	H(A2,A7)	0.0627	H(A3,A7)	0.0428	H(A4,A7)	0.0744
H(A1,A8)	0.0002	H(A2,A8)	0.0277	H(A3,A8)	0.0005	H(A4,A8)	0.0277
H(A1,A9)	0.0125	H(A2,A9)	0.0176	H(A3,A9)	0.0428	H(A4,A9)	0.0294
H(A1,A10)	0.0652	H(A2,A10)	0.0061	H(A3,A10)	0.0000	H(A4,A10)	0.0000
**A5**		**A6**		**A7**		**A8**	
H(A5,A1)	0.0345	H(A6,A1)	0.0043	H(A7,A1)	0.0000	H(A8,A1)	0.0129
H(A5,A2)	0.0631	H(A6,A2)	0.0447	H(A7,A2)	0.0512	H(A8,A2)	0.2475
H(A5,A3)	0.1265	H(A6,A3)	0.0705	H(A7,A3)	0.0724	H(A8,A3)	0.1804
H(A5,A4)	0.0453	H(A6,A4)	0.0349	H(A7,A4)	0.0573	H(A8,A4)	0.1589
H(A5,A6)	0.0357	H(A6,A5)	0.0223	H(A7,A5)	0.0080	H(A8,A5)	0.1194
H(A5,A7)	0.0690	H(A6,A6)	0.0288	H(A7,A6)	0.0086	H(A8,A6)	0.1204
H(A5,A8)	0.0277	H(A6,A8)	0.0019	H(A7,A8)	0.0000	H(A8,A7)	0.1151
H(A5,A9)	0.0176	H(A6,A9)	0.0076	H(A7,A9)	0.0000	H(A8,A9)	0.0356
H(A5,A10)	0.0116	H(A6,A10)	0.0094	H(A7,A10)	0.0231	H(A8,A10)	0.0816
**A9**		**A10**					
H(A9,A1)	0.0084	H(A10,A1)	0.0462				
H(A9,A2)	0.1830	H(A10,A2)	0.1270				
H(A9,A3)	0.1847	H(A10,A3)	0.0895				
H(A9,A4)	0.1470	H(A10,A4)	0.0273				
H(A9,A5)	0.0632	H(A10,A5)	0.0677				
H(A9,A6)	0.1039	H(A10,A6)	0.0448				
H(A9,A7)	0.0844	H(A10,A7)	0.1069				
H(A9,A8)	0.0014	H(A10,A8)	0.0282				
H(A9,A10)	0.0784	H(A10,A9)	0.0604				

**Table 7:** The value of net flow.

Area	2014	2015	2016	2017
Shanghai	0.6043	0.2353	0.3556	0.2580
Nanjing	−0.7700	−0.6263	−0.4744	−0.4290
Changzhou	−0.7629	−1.2823	−0.1018	−0.1068
Wuxi	−0.3033	−0.1555	−0.2793	−0.6038
Suzhou	−0.0141	−0.2096	−0.5179	−0.4044
Zhenjiang	−0.2306	0.0952	−0.7304	−0.7966
Hangzhou	−0.4334	−0.3318	−0.3339	−0.3186
Ningbo	0.9564	0.8795	0.7972	0.9109
Wenzhou	0.6309	0.7127	0.6533	0.5207
Shaoxing	0.3228	0.6828	0.6316	0.9695
